# Distinguishing subtypes of endothelial cells in the mouse aorta

**DOI:** 10.1073/pnas.2525755122

**Published:** 2025-12-04

**Authors:** Liqun He, Riikka Pietilä, Yuyang Miao, Elisa Vazquez-Liebanas, Marie Jeansson, Loren G. Fong, Stephen G. Young, Maarja Andaloussi Mäe, Lars Muhl, Christer Betsholtz

**Affiliations:** ^a^Department of Immunology, Genetics and Pathology, Rudbeck Laboratory, Uppsala University, Uppsala SE-751 85, Sweden; ^b^Department of Medicine-Huddinge, Karolinska Institutet, Huddinge SE-141 57, Sweden; ^c^Department of Medicine, David Geffen School of Medicine, University of California Los Angeles, Los Angeles, CA 90095; ^d^Centre for Cancer Biomarkers, Department of Clinical Medicine, University of Bergen, Bergen 5020, Norway

**Keywords:** aorta, endothelial cell, single-cell RNA sequencing

## Abstract

This study reanalyzed endothelial cell (EC) gene expression in the mouse aorta utilizing eight single-cell RNA-sequencing datasets. Contrary to the assumption that all ECs originated from the luminal surface, we identified two distinct sites of origin: aortic lumen (*Cytl1*-positive ECs) and peri-aortic microvascular (*Gpihbp1*-positive ECs). The proportions of these EC subtypes varied extensively across the eight datasets, likely due to differing experimental procedures. We deduced complete transcriptomes for each EC subtype, revealing differential gene expression and predicted functional properties. We provide marker lists and an accessible online database for gene-by-gene analysis to aid in correct cell identification and in gauging the impact of patho/physiological parameters on aortic EC gene expression.

## Significance

This study refines our understanding of the cells lining the mouse aorta, a blood vessel commonly used to model atherosclerotic disease. Using advanced single-cell RNA-sequencing analysis, distinct subtypes of endothelial cells (ECs) were identified based on their location: those lining the aortic lumen and those in the surrounding microvasculature. These EC subtypes exhibit significant differences in gene expression, suggesting specialized roles in lipid transport, metabolism, and vascular function. Previous work had assumed all these EC subtypes were found lining the aortic lumen, which this study demonstrates is not the case. This insight is crucial for future research on aortic diseases, enabling more precise identification and genetic targeting of specific EC subtypes.

Endothelial cells (ECs) lining the aortic lumen are critical players in vascular health and disease, directly exposed to factors like hypertension, shear stress, and atherogenic lipoproteins. While smooth muscle cells (SMCs), fibroblasts, and macrophages are established contributors to aorta atherogenesis, the role of ECs is, however, less understood (1, 2). A detailed characterization of aortic lumen ECs, distinct from ECs of other aortic locations, such as microvessels in the vascular adventia and adjacent surrounding tissue, is therefore essential.

Eight previous single-cell RNA-sequencing (scRNA-seq) studies of adult mouse aortas (3–10) have reported on gene expression patterns in aortic ECs and their correlations with various features, including cell proliferation (9), lipid metabolism (5), cell senescence (8), extracellular matrix production (5), vascular tone (8), endothelial–mesenchymal transition (5), inflammation (11), macrophage–endothelial cooperation (7), and receptor signaling (6). Moreover, relationships have been proposed between aortic EC gene expression and pathophysiological challenges such as aging (8), hypertension (9), diabetes mellitus (9), high-fat diet (11), and atherosclerosis (10). In each study, the underlying assumption was that the aortic ECs originated from the luminal surface. However, because our previous work uncovered significant EC heterogeneity related to arterio-venous zonation (12–14), we suspected that the ECs were from different aortic locations; therefore, we reanalyzed the scRNA-seq data from the eight studies.

## Results

### Distinguishing Markers for Mouse Aortic Luminal and Peri-Aortic ECs.

**Fig. 1.**
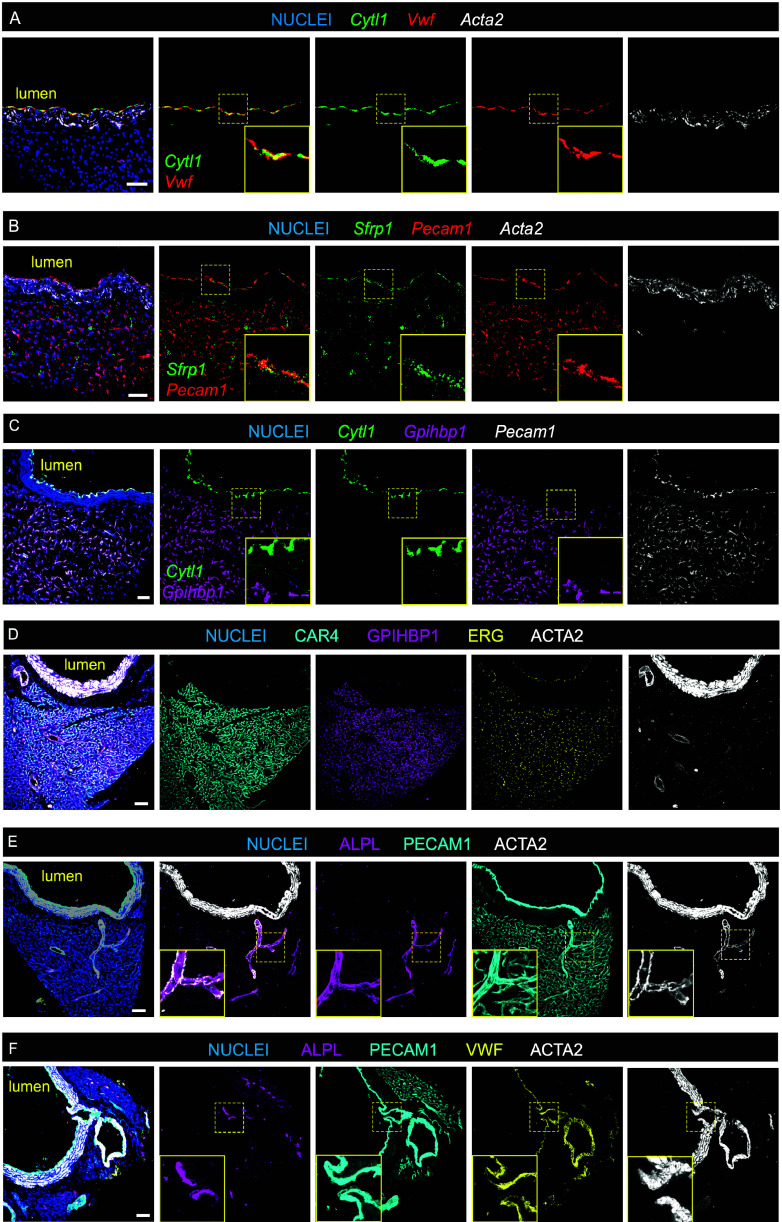
Distinguishing markers for aortic luminal ECs and peri-aortic adipose tissue ECs in the adult mouse aorta. (*A*) ISH analysis shows that *Cytl1* and *Vwf* mRNAs colocalize in aortic luminal ECs (*Inset*). *Acta2* was used to label SMCs. Nuclei were stained with DAPI. (*B*) ISH analysis shows *Sfrp1* expression in luminal EC colocalized with the general EC marker *Pecam1* (*Inset*). Sporadic *Sfrp1+* cells in the peri-aortic tissue probably reflect *Sfrp1* expression in fibroblasts. (*C*) ISH show *Gpihbp1* expression in peri-aortic capillary ECs but not in *Cytl1-*positive luminal EC (*Inset*). (*D*) IF analysis shows that CAR4 and GPIHBP1 colocalize in peri-aortic capillary ECs and are absent in aortic luminal ECs. ERG IF visualizes EC nuclei. Hoechst stains nuclei. (*E*) IF analysis shows that ALPL expression in the peri-aortic vasculature localizes to ACTA2-positive arterial/arteriolar branches (*Inset*), as well as to ACTA2 weak/negative microvessels. No ALPL positive ECs were observed in the aortic luminal EC. (*F*) IF analysis at an intercostal artery branch point with antibodies for ALPL, PECAM1, VWF, and ACTA2. ALPL expression labels specifically EC at the branch point junction site (*Inset*). VWF IF labels the aortic lumen EC. (Scale bar: 50 μm.)

A minimum number of known distinguishing markers is required for cell type annotation of scRNA-seq data. To this end, we analyzed adult mouse aortas using in situ hybridization (ISH) and immunofluorescence (IF) for selected mRNA and protein markers of large and small vessel ECs (15, 16). This revealed that only luminal ECs were uniformly positive for *Cytl1* and *Sfrp1*, whereas only microvascular ECs in adjacent peri-aortic tissue (predominantly brown adipose tissue) were positive for *Gpihbp1* and CAR4 (Fig. 1 *A*–*F*). ALPL was missing in luminal ECs but expressed in capillary, arteriolar, and arterial branch point ECs (Fig. 1 *F* and *G*).

### Reanalysis of Mouse Aorta scRNA-seq Data.

**Fig. 2.**
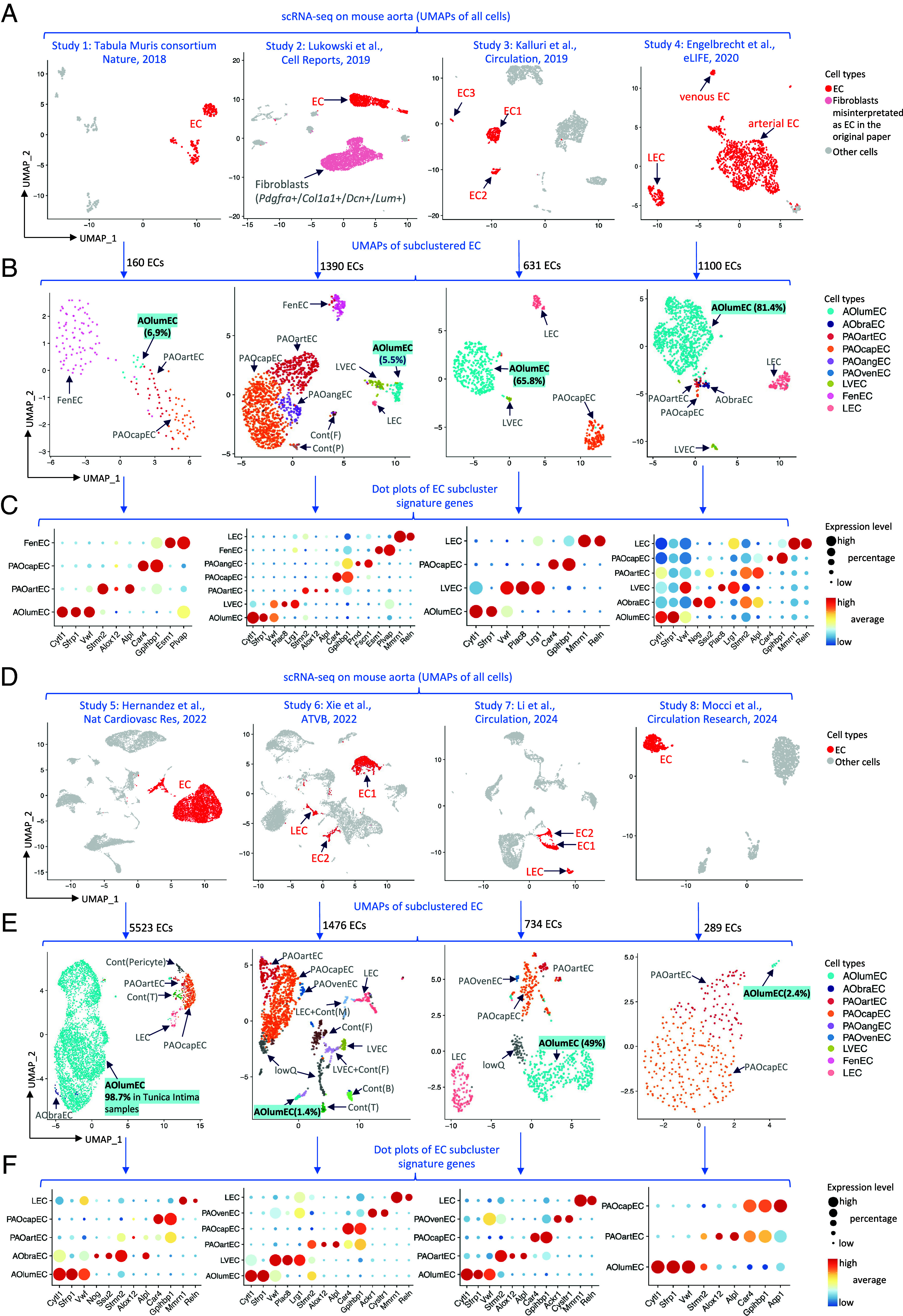
Mouse aorta single-cell RNA sequencing, EC subclustering, annotation, and validation. (*A*) UMAP overview of data from studies 1 to 4. (*B*) ECs from A were subclustered. AOlumEC, aortic luminal EC; AObraEC, aortic intercostal branch point EC; PAOartEC, peri-aortic arteriolar EC; PAOcapEC, peri-aortic capillary EC; PAOvenEC, peri-aortic venular EC; PAOangEC, peri-aortic angiogenic EC; LEC, lymphatic EC; LVEC, large vein (azygous vein or vena cava) EC; FenEC, fenestrated EC; Cont(x), contamination(cell type); F, fibroblasts; P, platelets; M, macrophages; T, T-cells; B, B-cells; lowQ, low quality transcriptomes. (*C*) Dot plots illustrating the expression of EC subtype markers. (*D*–*F*) Same analysis as shown in *A*–*C* for studies 5 to 8.

Fig. 2 illustrates our scRNA-seq reanalysis of the eight previous studies (3–10). We first confirmed the basal endothelial identity of the reported EC clusters in each individual study (Fig. 2 *A* and *D*). Endothelial cells were identified using canonical markers, including *Pecam1, Cdh5, Fli1, Tek, Ptprb*, and others. This confirmed the authors’ annotations in all studies, with the exception of Study-2 (4), in which one cell cluster claimed to be endothelial cells was identified as fibroblasts based on the combined low abundance or absence of canonical endothelial markers and the presence of canonical fibroblast markers, such *as Pdgfra, Col1a1, Lum, Dcn*, and other markers of fibroblasts identified previously (17) (Fig. 2*A*). The originally reported names of the EC subtypes for each study are shown in Fig. 2 *A* and *D*.

We next subclustered the EC transcriptomes from each of the eight studies (Fig. 2 *B* and *E*) and assigned distinguishing gene signatures for the subclusters (Fig. 2 *C* and *F*). Only one subcluster expressed *Cytl1* and *Sfrp1,* consistent with aortic lumen ECs. We concluded that the other subclusters represented venous (expressing e.g., *Lrg1*, *Ackr1*), capillary (e.g., *Car4, Gpihbp1*), fenestrated (e.g., *Plvap, Esm1*), or lymphatic (e.g., *Reln, Mmrn1*) ECs based on their marker profiles. The reader may assess and further explore the aortic endothelial differential gene expression patterns gene-by-gene across the individual studies (3–10) through the searchable database available at https://betsholtzlab.org/Publications/MouseAortaEC/database.html. The fibroblast cluster in Study-2 can be explored gene-by-gene in an interactive online database available at https://heomics.shinyapps.io/Mouse_Aorta_scRNAseq_CellReport2019/.

In one study (Study 5), lumen ECs represented 98.7% of the aortic ECs. In the other studies (3–6, 8–10), the proportion of *Cytl1*-positive ECs ranged from 1.4 to 81.4% (Fig. 2). Conversely, the proportion of *Gpihbp1*-positive microvascular ECs ranged from 14.0 to 97.6% (Fig. 2 *B* and *E*). Apparently, capillary, lymphatic, venous, and fenestrated ECs were contributed by peri-aortic adipose tissue, lymphatics, and veins that remained attached to the dissected aortas to varying degrees in the different studies.

### Differentially Expressed Gene (DEG) Analysis between Aortic Lumen and Peri-Aortic ECs.

**Fig. 3.**
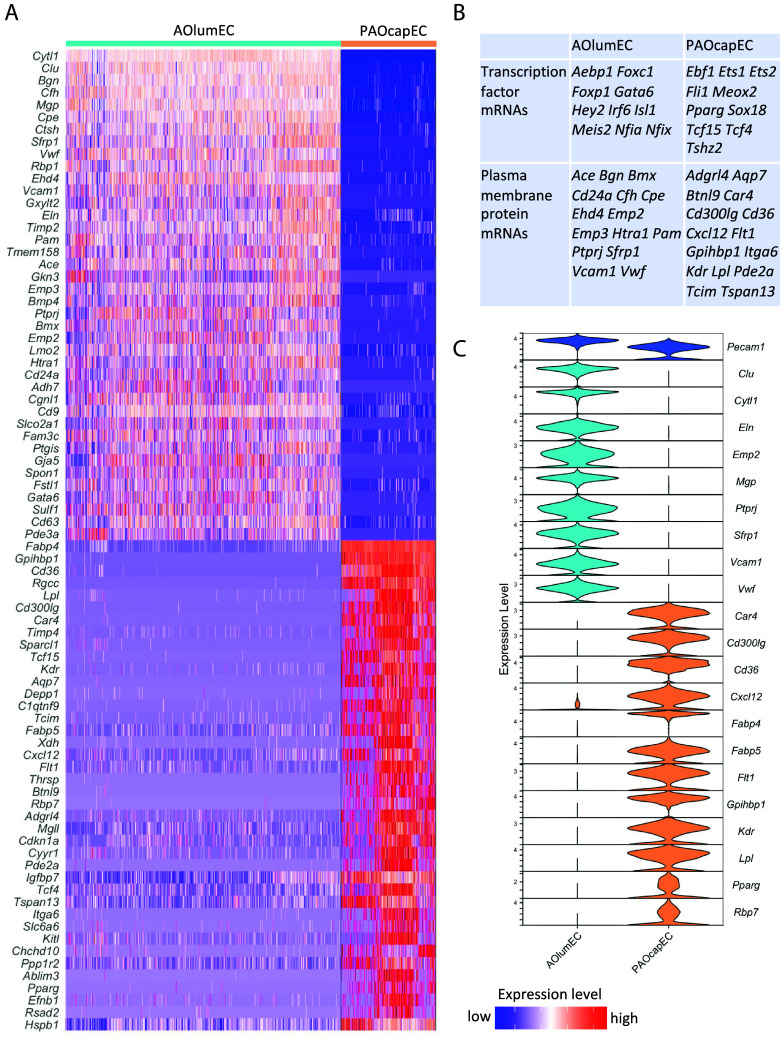
DEGs between aortic lumen ECs and peri-aortic capillary ECs. (*A*) Heatmap overview of the top 80 DEGs. (*B*) List of transcription factors and top plasma membrane protein DEGs. (*C*) Violin plot of gene transcripts mentioned in the text.

We next deduced DEGs between luminal and peri-aortic microvascular ECs (Fig. 3). Notably, several top DEGs enriched in peri-aortic microvascular ECs encode proteins involved in lipid transport and metabolism (*Cd300lg*, *Cd36*, *Fabp4*, *Fabp5*, *Gpihbp1*, *Lpl*, *Pparg*, *Rbp7*) and angiogenesis (*Cxcl12*, *Flt1*, *Kdr*), whereas the top aortic luminal EC-enriched DEGs encode proteins implicated in, e.g., calcium signaling (*Clu*, *Ptprj*, *Vcam1*), matrix adhesion (*Emp2*, *Sfrp1*), and elastic properties (*Eln, Mgp*). These differences likely reflect the different physiological roles of ECs in capillaries vs. large elastic arteries.

## Discussion

Our reanalysis of eight previous scRNA-seq studies of the mouse aorta shows that the ECs assumed to be of luminal location in fact represent a mixture of ECs from different anatomical locations within and around the aorta. In several of the studies (4–6, 8–10), conclusions were drawn about the influence of physiological and environmental variables on aortic lumen ECs without recognizing that a significant portion of these ECs, in some cases the majority, originated from other sources. This oversight potentially invalidates or casts doubt on many of those conclusions. The variation in the proportions of luminal and peri-aortic microvascular ECs observed across the eight studies likely resulted from technical factors. Skewed representation of cell types is a common limitation in scRNA-seq of solid tissues, often caused by differences in cell fragility and dissociation efficiency. The high recovery rate of luminal ECs in Study 5 (98.7%) was achieved by initially scraping the endothelial layer from the aortic surface before generating single-cell suspensions (7).

The distinguishing markers identified here should enable accurate cell identification and facilitate more precise analyses of how physiologic, pathophysiologic, and environmental perturbations affect gene expression in aortic luminal and peri-aortic ECs. Additionally, the reported gene expression differences are likely to aid in developing new tools for the specific targeting of EC subtypes, such as antibodies for cell panning and Cre drivers for genetic recombination.

## Materials and Methods

### Animals.

All mouse experiments were conducted in accordance with Swedish and European legislation. Ethical permits were approved by the Animal Ethics Committee in Uppsala (C115/15, 5.8.18-03029/2020). Animals were housed on a 12-h light–dark cycle, with ad libitum access to water and standard chow. For all experiments, adult (>8-wk) C57BL6/J mice (from in-house breeding) of both sexes were used.

### Mouse Aorta Tissue Preparation for Protein and mRNA In Situ Detection.

For IF microscopy, mouse thoracic aortae together with the surrounding periaortic adipose tissue depots were dissected and immersion fixed at room temperature in either 2% or 4% (antibody dependent) formaldehyde solution (#02176, Histolab) for 3 to 4 h. After washing with phosphate-buffered saline (PBS; #14190, Gibco), tissues were embedded in Optimum Cutting temperature compound Neg-50 (#6502, Epredia) and stored at –80 °C. For RNAscope™ studies, samples were dissected as described earlier, snap-frozen in Neg-50 medium without any prior fixation, and stored at –80 °C. Samples were cut into cryosections with a cryotome (Cryostar NX70, Thermo Fisher) and mounted on Superfrost™Plus Adhesion microscope slides (Epredia) and stored at –80 °C.

### IF Microscopy.

20-µm-thick cryosections were let to stand at room temperature for 15 min before washing twice with PBS. Samples were incubated 3 to 4 h in 1× blocking/permeabilization solution (0.5% Triton-X 100 in Protein Block; DAKO, X0909). Primary antibodies were diluted in 0.5× blocking/permeabilization solution and incubated overnight at 4 °C. After washing three times with PBS, samples were incubated at RT for 2 to 3 h in a secondary antibody solution (in 1× PBS). Samples were washed three times with PBS (second wash with 1:10,000 Hoechst #33342, Invitrogen; in PBS to label the nuclei) before mounting in ProLong Gold Antifade reagent (Invitrogen). Images were acquired with a Leica TCS SP8 confocal microscope with LAS X software (version 3.5.723225, Leica Microsystems).

The following antibodies were used: ALPL (#AF2910, R&D Systems; 1:200), FITC-conjugated α-SMA (#F3777, Sigma; 1:200), CAR4 (#AF2414, R&D Systems; 1:200), ERG (#ab92513, Abcam; 1:200), mAb 11A12 against GPIHBP1 (own production, 1:300), PECAM1 (#AF3628, R&D systems; 1:300) PECAM1 (#553370, BD Biosciences; 1:100), vWF (#A0082, DAKO; 1:500). Secondary antibodies (all from donkey) were anti-rabbit CF™633 (#SAB4600132, Sigma-Aldrich), anti-goat AlexaFluor™680 Plus (#A32860, Thermo Fisher), anti-rat AlexaFluor™555 Plus (#A48270, Thermo Fisher), anti-goat AlexaFluor™633 (Thermo Fisher), and anti-rabbit AlexaFluor™680 (Nordic Biosite).

### In Situ RNA Hybridization Studies.

RNAscope™ Multiplex Fluorescent Reagent Kit (v.2) (Advanced Cell Diagnostics, United States) and TSA Plus reagents (Perkin Elmer) were used according to the manufacturer’s protocol for fresh-frozen sections with minor modifications. Briefly, 20-μm cryosections were fixed with 4% buffered formaldehyde (#02176, Histolab) for 45 min at 4 °C and rinsed twice with PBS. Pretreatment was performed by dehydration with ethanol (50%, 70%, and twice with 100%) for 5 min at room temperature; subsequently, slides were processed immediately or stored in 100% EtOH at –20 °C for up to 1 wk. Sections were dried and a hydrophobic barrier was created using Immedge™ Hydrophobic Barrier Pen (Vector Laboratory, United States). Autofluorescence was quenched with a Bloxall™ blocking solution (Vector laboratories) for 10 min at room temperature; sections were then rinsed twice with water and permeabilized for 20 min with Pretreat III. After rinsing twice with PBS, 100 μL of probe mix was applied to sections and were incubated for 2 h at 40 °C. Fluorescent signals were developed and amplified according to the manufacturer’s protocol and then mounted in ProLong Gold Antifade reagent (Invitrogen). RNAscope probes (all from ACDBio) were *Acta2* (319531), *Cytl1* (817241-C2), *Gpihbp1* (540631-C3), *Sfrp1* (404981-C2), *Pecam1* (316721, or 316721-C3), and *Vwf* (499111-C3).

### scRNA-seq Data Analyses.

The raw sequence data for the eight aortic studies (3–10) were obtained from NCBI GEO database (https://www.ncbi.nlm.nih.gov/geo), ArrayExpress (https://www.ebi.ac.uk/arrayexpress), or AWS server (https://registry.opendata.aws). The accession names or numbers are tabula-muris-senis (AWS, Study-1) (3), E-MTAB-7148 (ArrayExpress, Study-2) (4), GSE174384 (GEO, Study-3) (5), GSE139065 (GEO Study-4) (6), GSE161787 (GEO, Study-5) (7), GSE164585 (GEO Study 6) (8), GSE234462(GEO, Study-7) (9), and GSE260656 (GEO, Study-8) (10). In Study-6, only the 4-wk-old samples were included in our reanalysis. In Study-8, only the wild-type control samples were included.

The raw sequence data were aligned to mouse genome assembly mm10 (refdata-gex-mm10-2020-A). The 10x data were aligned and summarized with CellRanger (version 4.0.0) using default settings. The Smart-seq2 data were aligned with Star software (version 2.7.3a). Samtools software (version 1.16.1) was used to filter out the duplicated reads, and the Subread package (version 1.4.6-p5) was used to summarize the gene counts with the featureCounts function. For each dataset, the raw gene counts data were processed in R Seurat packages (version: 4.3.0) for quality control, normalization, scaling, clustering, and further downstream visualizations. The cells with less than 500 detected genes or mitochondrial gene percentage over 10% were excluded. A scale factor of 10,000 reads per cell was used to normalize each cell. For dimensional reduction, the Uniform Manifold Approximation and Projection (UMAP) method in Seurat package was utilized. The UMAP and dot-plot were visualized with the Seurat DimPlot and FeaturePlot functions. The interactive Shiny app with online database was constructed using the ShinyCell package (version: 2.1.0).

To identify DEGs between aortic luminal EC (AOlumEC) and peri-aortic capillary EC (PAOcapEC), the Seurat FindMarkers function was used (on cells from all five 10× datasets, including 5,760 AolumEC and 1,960 PAOcapEC). This function first applies the Wilcoxon rank sum test to assess differential expression, followed by multiple testing correction with the Bonferroni method. A corrected *P*-value threshold of <0.05 was applied to identify genes with significant differential expression. The top 80 DEGs were visualized with the Seurat DoHeatmap function (Fig. 3*A*). Here, the cells were ordered according to study resulting in vertical stripes in the heat pattern correlating with study origin. These differences (stripes) probably reflect batch effects. Violin plots were generated using Seurat’s VlnPlot function to illustrate the differences in gene expression between the two groups. To account for unequal cell numbers, 1,000 cells were randomly selected from each group for visualization.

To identify transcription factor- and plasma membrane protein–encoding genes (Fig. 3*B*) we made use of Gene Ontology (GO) annotation (www.geneontology.org) and utilized R package GO.db (version 3.16.0) and org.Mm.eg.db (version 3.16.0). All genes annotated as belonging to DNA-binding transcription factor activity (GO:0003700) and plasma membrane (GO:0005886) were extracted. GO enrichment analysis for the most enriched genes in peri-aortic capillary EC and aortic lumen EC were performed with R package enrichR (version 3.1), which applies a hypergeometric test, followed by multiple testing correction with the False Discovery Rate method. A significance threshold of adjusted *P*-value < 0.05 was used.

## Data Availability

A searchable database of aortic EC subtype scRNA-seq data is available online: https://betsholtzlab.org/Publications/MouseAortaEC/database.html (18). Previous published data (refs. 3–10) were reanalyzed for this work. All other data are included in the main text.
